# Hepatocyte Growth Factor Activator Inhibitor-1 Is Induced by Bone Morphogenetic Proteins and Regulates Proliferation and Cell Fate of Neural Progenitor Cells

**DOI:** 10.1371/journal.pone.0056117

**Published:** 2013-02-07

**Authors:** Raili Koivuniemi, Johanna Mäkelä, Marie-Estelle Hokkanen, Céline Bruelle, Tho Huu Ho, Roxana Ola, Laura Korhonen, Jim Schröder, Hiroaki Kataoka, Dan Lindholm

**Affiliations:** 1 Institute of Biomedicine/Biochemistry and Developmental Biology, University of Helsinki, Helsinki, Finland; 2 Minerva Foundation Institute for Medical Research, Biomedicum-2 Helsinki, Helsinki, Finland; 3 Neuroscience Center, University of Helsinki, Helsinki, Finland; 4 Department of Pathology, Faculty of Medicine, University of Miyazaki, Miyazaki, Japan; Duke University Medical Center, United States of America

## Abstract

**Background:**

Neural progenitor cells (NPCs) in the developing neuroepithelium are regulated by intrinsic and extrinsic factors. There is evidence that NPCs form a self-supporting niche for cell maintenance and proliferation. However, molecular interactions and cell-cell contacts and the microenvironment within the neuroepithelium are largely unknown. We hypothesized that cellular proteases especially those associated with the cell surface of NPCs play a role in regulation of progenitor cells in the brain.

**Methodology/Principal Findings:**

In this work, we show that NPCs, isolated from striatal anlage of developing rat brain, express hepatocyte growth factor activator inhibitor-1 and -2 (HAI-1 and HAI-2) that are cell surface-linked serine protease inhibitors. In addition, radial glia cells derived from mouse embryonic stem cells also express HAI-1 and HAI-2. To study the functional significance of HAI-1 and HAI-2 in progenitor cells, we modulated their levels using expression plasmids or silencing RNA (siRNA) transfected into the NPCs. Data showed that overexpression of HAI-1 or HAI-2 decreased cell proliferation of cultured NPCs, whilst their siRNAs had opposite effects. HAI-1 also influenced NPC differentiation by increasing the number of glial fibrillary acidic protein (GFAP) expressing cells in the culture. Expression of HAI-1 *in vivo* decreased cell proliferation in developing neuroepithelium in E15 old animals and promoted astrocyte cell differentiation in neonatal animals. Studying the regulation of HAI-1, we observed that Bone morphogenetic protein-2 (BMP-2) and BMP-4 increased HAI-1 levels in the NPCs. Experiments using HAI-1-siRNA showed that these BMPs act on the NPCs partly in a HAI-1-dependent manner.

**Conclusions:**

This study shows that the cell-surface serine protease inhibitors, HAI-1 and HAI-2 influence proliferation and cell fate of NPCs and their expression levels are linked to BMP signaling. Modulation of the levels and actions of HAI-1 in NPCs may be of a potential value in stem cell therapies in various brain diseases.

## Introduction

Interactions between proteases and their inhibitors play an important role in development and post-injury tissue remodeling. Particularly proteases linked to the cell surface and the pericellular space are crucial for cell-cell contacts and interactions with the extracellular matrix [1], [2]. In the brain, NPCs are present in the developing neuroepithelium in a local microenvironment and form a self-supporting niche that regulates cell maintenance and proliferation [3]. In this local tissue milieu the stem and progenitor cells can be in contact with other cell types such as endothelial cells and immature neuroblasts and glial cells [2], [3]. The mechanism governing the interactions between these different cells types is largely unknown but may involve proteases and their inhibitors. It is also known that NPCs *in vitro* grow preferentially as neurospheres suggesting that cell-cell contacts and surface interactions are important for their development. However, apart from cell adhesion molecules and integrins little is known about cell surface-associated proteins and how they influence NPCs. In this study, we have focused on the expression of cell-surface linked protease inhibitors in the NPCs and whether these putative molecules might influence cell proliferation or differentiation of the NPCs.

Hepatocyte growth factor activator inhibitor-1 (HAI-1) and -2 (HAI-2) are type I transmembrane glycoproteins that belong to the Kunitz type serine protease inhibitor family, and they are expressed by epithelial cells in all major organs of the body [4]–[6]. We therefore studied whether these molecules are also present in the neuroepithelium harboring the NPCs and their progeny. We observed that NPCs derived from developing rat striatum express HAI-1 and HAI-2 in cell culture as well as in developing rat neuroepithelium. We further noted that the modulation of the cell surface-expression of HAI-1 and HAI-2 had a robust effect on cell proliferation of NPCs. Particularly, HAI-1 exhibited effects on cultured rat NPCs increasing cell division and promoting glial cell differentiation. Overexpression of HAI-1 in the developing mouse brain in utero reduced cell proliferation in E14 old neuropeithelium and promoted astroglia formation in E17 to P1 old neuroepithelium. Studies in cell culture showed that the expression of HAI-1 and HAI-2 is increased by BMP-2 and BMP-4 acting via the BMP receptors, BMPR-IA and BMPR-IB (also called ALK-3 and ALK-6 receptors, respectively) that are expressed in developing progenitor cells and in the developing neuroepithelium. This study shows that there is a link between the action of growth factors such as BMP-2 and the serine protease inhibitor HAI-1 in the NPCs and that this can contribute to the regulation of these progenitor cells and the local tissue milieu in the developing brain.

## Results

### NPCs Express the Cell-surface Proteins HAI-1 and HAI-2

**Figure 1:**
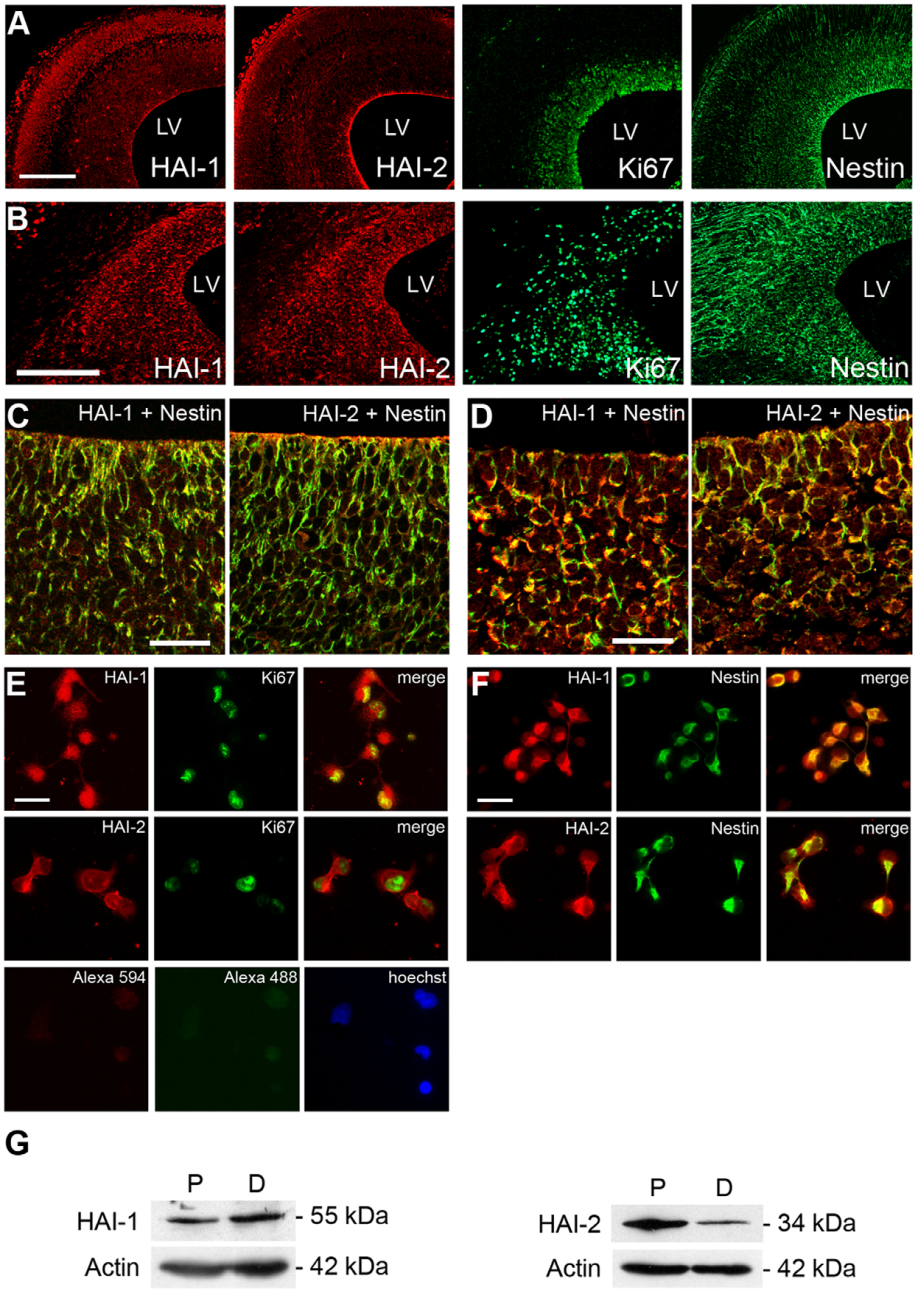
HAI-1 and HAI-2 are expressed in neural progenitor cells and in the developing neuroepithelium. Sections were made from dorsal area of developing embryonic day 17 (E17)-old and postnatal P1-day old rat brain cortex. Immunostaining was done using specific antibodies as described in Methods. Neural progenitor cells were prepared from the striatal anlage of E17 old rats and cultivated as described in Methods. Nestin is a marker for progenitor cells and Ki67 for dividing cells. (**A–B**) Immunofluorescence of dorsal cortex of E17 old (A) or P1 old rats (B). Left panels, HAI-1 and HAI-2 positive cells (red fluorescence). Right panels, Ki67- and Nestin-positive cells (green fluorescence). LV, lateral ventricle. Scale bar, 200 µm. (**C–D**) Higher magnification of confocal images showing double-staining of HAI-1 and HAI-2 (red fluorescence) with Nestin positive cells (green fluorescence) in E17 (C) and P1 (D) brain. Scale bars, 50 µm. (**E–F**) Immunocytochemistry of NPCs isolated from developing striatum of E17 rat brain. HAI-1 and HAI-2 are shown in red and Ki67 (E) and Nestin (F) in green fluorescence. Merged pictures to the right show double-stained cells (yellow fluorescence). Note double staining of almost all Nestin-positive cells. Lower panels in (E) show control staining without secondary antibodies, and nuclei are stained using Hoechst blue. Scale bars, 20 µm. (**G**) Immunoblots of the NPCs cultivated under proliferating (P) or differentiating (D) conditions (see Methods). HAI-1 (55 kDa band) remains fairly constant, whereas HAI-2 (34 kDa) decreases during cell differentiation. ß-actin was used as a loading control.

In this study, we observed that HAI-1 and HAI-2 are expressed by nestin-positive NPCs in the embryonic (E17) and in the postnatal (P1) old rat neuroepithelium, as shown by immunostaining of dorsal cortex sections (Fig. 1A–D). Data obtained in culture showed that NPCs derived from E17 old rat striatum also express HAI-1 and HAI-2 (Fig. 1E–F). Double staining revealed that most of the HAI-1 and HAI-2-labeled cells are positive for nestin identifying them as NPCs (Fig. 1F). Likewise many cells positive for HAI-1 or HAI-2 were also labeled with Ki67 as a marker for dividing cells (Fig. 1E). Control experiments omitting the first antibodies showed no immunostaining of cells (Fig. 1E). Immunoblots of cell lysates showed that HAI-1 and HAI-2 are present in cells grown as neurospheres and in cells undergoing differentiation (Fig. 1G). This data shows that HAI-1 and HAI-2 are expressed by NPCs, raising questions about their putative functions.

### HAI-1 and HAI-2 Decrease Cell Proliferation of NPCs

**Figure 2:**
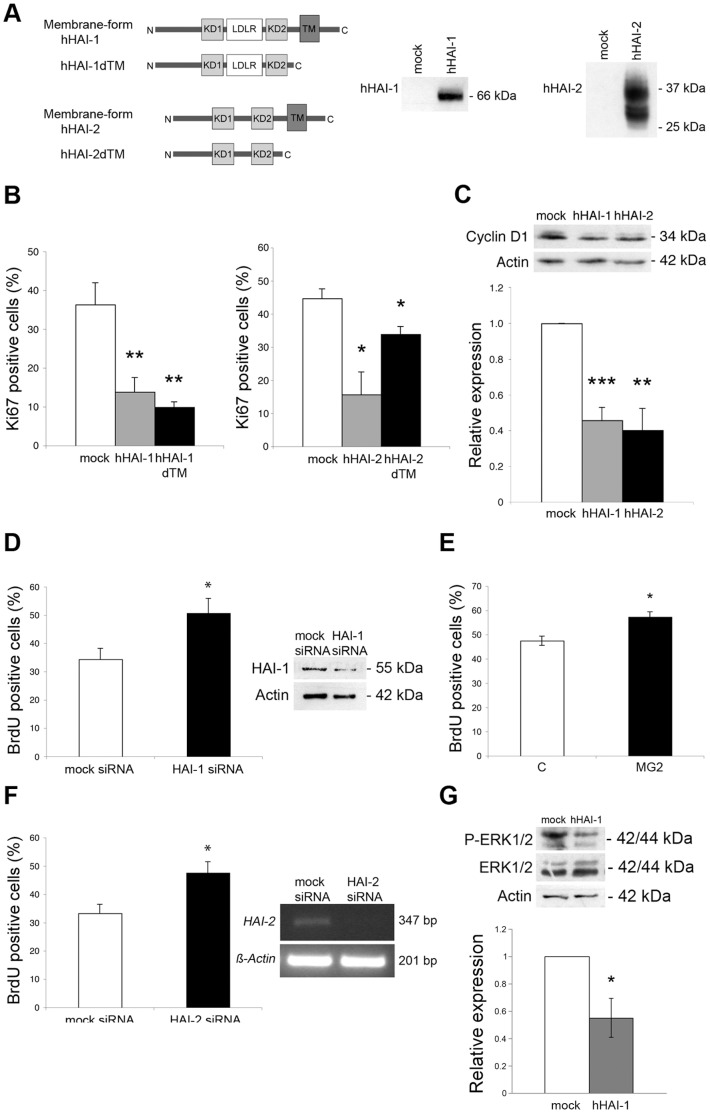
Effects of HAI-1 and HAI-2 overexpression and downregulation on cell proliferation in NPCs. NPCs were isolated from developing E17-old striatum and were transfected as described in Methods using overexrepressing constructs or silencing RNA (siRNA) as indicated. (**A**) Left, Schematic view of human full-length and transmembrane-lacking (dTM) constructs of HAI-1 and HAI-2. 8 µg of expression plasmids for these constructs were transfected into NPCs as described in Methods. Control cells were transfected with EGFP plasmids. Right, Immunoblots of transfected cells detected using anti-HAI-1 and anti-HAI-2 antibodies detecting human HAI-1 at 66 kDa, and human HAI-2 at 34 kDa and 28 kDa, respectively. N, aminoterminus; KD, Kunitz domain; LDLR, LDL receptor-like domain; TM, transmembrane domain; C, carboxyterminus. (**B**) The number of Ki67-positive of all cells was determined by immunocytochemistry. Expression of HAI-1 and HAI-2 for 6 days reduced the number of dividing cells compared with controls (mock). Values are means ± SEM, n = 3. **p<0.01 for HAI-1 constructs vs. control, and *p<0.05 for HAI-2 constructs vs. control. (**C**) Immunoblot of the cell cycle regulator, CyclinD1. ß-actin was used as a control. Quantification below shows a decrease in CyclinD1 after HAI-1 and HAI-2 expression. Values are means ± SEM, n = 4. ***p<0.001 for HAI-1 vs. control, and **p<0.01 for HAI-2 vs. control. (**D**) Downregulation of HAI-1 by siRNA increased the number of dividing cells. Control cells received scrambled siRNA. After 3 days with siRNA the number of BrdU-positive cells was determined by immunostaining. Values are means ± SEM, n = 4. *p<0.05 for HAI-1 siRNA vs. control. HAI-1 levels are shown to the right. (**E**) Cell culture supernatant from hybridoma cells producing monoclonal anti-HAI-1 antibodies (MG2, see Hallikas et al., 2006) was diluted 1∶50 and added to the cultures for 3 days. Note an increase in the number of dividing NPCs. Values are means ± SEM, n = 3. *p<0.05 for MG2 vs. control. (**F**) Downregulation of HAI-2 by siRNA increased the number of BrdU-positive dividing cells after 3 days. Control cells received scrambled siRNA. Values are means ± SEM, n = 3. *p<0.05 for HAI-2 siRNA vs. control. HAI-2 mRNA levels are shown to the right. (**G**) Immunoblot of Erk1/2. The phosphorylation of Erk1/2 was reduced by HAI-1 overexpression. Total Erk1/2 was not changed. ß-actin was used as a control. Quantification below. Values are means ± SEM, n = 3. *p<0.05 for HAI-1 vs. control.

To study whether HAI-1 and HAI-2 influence NPCs we used either overexpressing plasmids, as depicted in Fig. 2A, or small interfering RNA (siRNA). Overexpression of HAI-1 and HAI-2 in the NPCs decreased the number of dividing cells, as shown by Ki67 immunostaining (Fig. 2B). HAI-1 and HAI-2 exist as membrane-bound and secreted proteins (Fig. 2A), and both isoforms significantly reduced the number of Ki67-positive cells (Fig. 2B). CyclinD1 is a major regulator of the cell cycle in developing NPCs [7], and the level of this protein was decreased by overexpression of HAI-1 and HAI-2, which is in line with the reduced cell proliferation (Fig. 2C). Furthermore, employing siRNAs to downregulate HAI-1 or HAI-2 increased the number of BrdU-positive cells (Fig. 2D and Fig. 2F). The addition of a blocking antibody against HAI-1 to the cultures [8] led to an increase in the number of proliferating NPCs (Fig. 2E).

### Alterations in Erk1/Erk2 Phosphorylation in NPCs by HAI-1 and HAI-2

The mechanism underlying the changes in cell proliferation observed with HAI-1 and HAI-2, probably involves alterations in cell signaling cascades subsequent to changes in cell-surface protease activity. Activation of the Ras/extracellular signal-regulated kinase-1 and -2 (Erk1/Erk2) is linked to increased progenitor cell proliferation [9]. We observed that the degree of phosphorylation of Erk1/Erk2 was decreased in the NPCs after HAI-1 expression (Fig. 2G). Studying the putative targets for HAI-1 and HAI-2, we first focused on HGFA, which is involved in hepatocyte growth factor (HGF) signaling. NPCs express HGF and HGFA but the high-affinity c-Met receptor for HGF was hardly detectable in NPCs under the present culture conditions (Fig. S1). Incubation of NPCs in the presence of the c-Met inhibitor, SU11274 did not influence the number of BrdU-positive cells nor did the addition of 20 ng/ml HGF (Fig. S1). These and other data suggest that HGF is not essentially involved in the HAI-mediated effects on cell proliferation in the NPCs.

### Stimulation with BMPs Increase the Expression of HAI-1 and HAI-2 in NPCs

**Figure 3:**
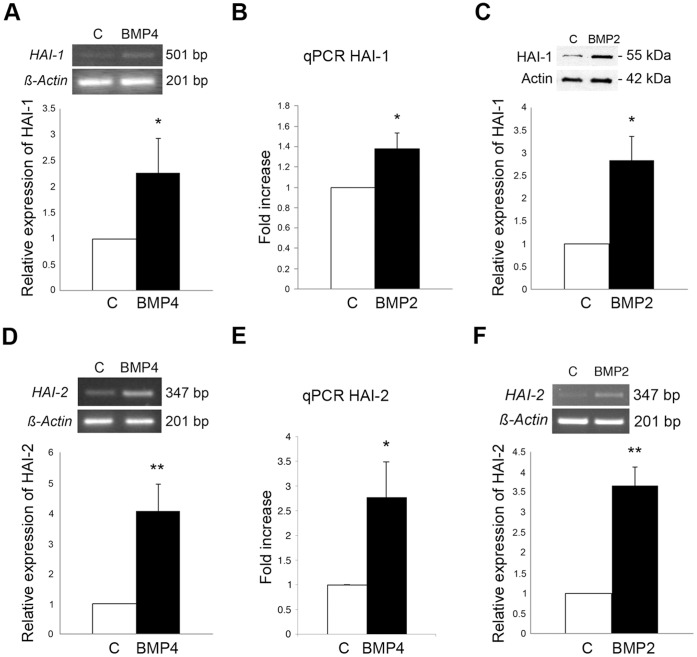
Bone morphogenetic proteins (BMPs) regulate HAI-1 and HAI-2 expression. NPCs were isolated from developing striatum of E17 old rats and treated with 100 ng/ml BMP-2 or BMP-4 and analyzed further as indicated. (**A–C**) HAI-1 changes. Expression of HAI-1 was analyzed using RT-PCR (A) and qPCR (B) after 24 h stimulation and by immunoblotting (C) after 3 days stimulation. Values are means ± SEM, n = 4. *p<0.05 for BMPs vs. control. (**D–F**) HAI-2 changes. Expression of HAI-2 was analyzed using RT-PCR (D, F) after 3 days of stimulation and by qPCR (E) after 24 h stimulation. Values are means ± SEM, n = 4. **p<0.01 and *p<0.05 for BMPs vs. control.

To search for putative factor(s) that regulate HAI-1/HAI-2, we stimulated NPCs with different growth factors followed by PCR analyses. Data showed that BMP-4 significantly increased the expression of HAI-1 (Fig. 3A) and HAI-2 in the NPCs (Fig. 3D and Fig. 3E), whereas transforming growth factor-β and Wnt-3 produced minor effects (data not shown). Similar effects as with BMP-4 was also observed using BMP-2 (Fig. 3B and Fig. 3F). This is in line with the fact that BMP-2 and BMP-4 share the same type of BMP type I receptors, BMPR-IA and BMPR-IB [10]. Using immunoblotting it was found that BMP-2 increased HAI-1 levels in the NPCs (Fig. 3C). Collectively this data shows that the BMP-2 and BMP-4 increase expression of the serine protease inhibitors, HAI-1 and HAI-2 in the NPCs.

### Role of HAI-1 and HAI-2 in the BMP-mediated Effects in NPCs

**Figure 4:**
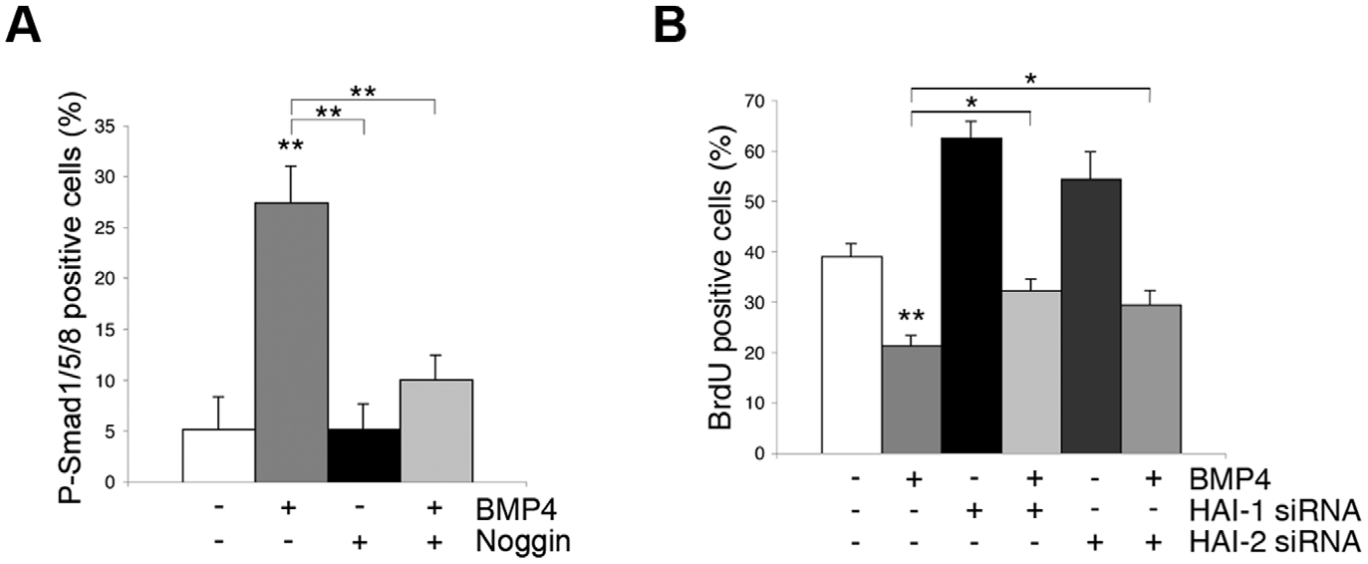
BMP-4 influences NPC division through HAI-1. NPCs were isolated from developing striatum of E17 old rats and treated as indicated below. (**A**) Immunostaining. Stimulation with 25 ng/ml BMP-4 increases the number of phospho-Smad positive NPCs and this was inhibited by adding 25 ng/ml Noggin. Values are means ± SEM, n = 3. **p<0.01 for BMP-4 vs. control and for BMP-4+Noggin vs. BMP-4. (**B**) siRNAs were added for 48 h to downregulate HAI-1 and HAI-2. Cells were further treated with 25 ng/ml BMP-4 for another 24 h, and the number of BrdU-positive cells determined. Values are means ± SEM, n = 3. **p<0.01 for BMP-4 vs. control and *p<0.05 for HAI-1/HAI-2 siRNA +BMP-4 vs. BMP-4 treatment.

Previously, BMPs have been shown to influence different progenitor cell populations in the brain [11]–[16]. Activation of the BMP receptors in target cells leads to a phosphorylation of Smad that can be studied using phospho-Smad (p-Smad) specific antibodies. We observed that there are few p-Smad immunopositive cells in the NPC cultures under control conditions (Fig. 4A), but this number increased significantly after stimulation with BMP-4 (Fig. 4A). Addition of Noggin that inhibits BMP signaling blocked this increase. We further observed that the addition of either 25 ng/ml BMP-4 (Fig. 4B) or BMP-2 (data not shown) decreased the number of BrdU-positive cells in the NPCs. Most importantly the decrease in cell division was significantly counteracted by the downregulation of either HAI-1 or HAI-2 using the corresponding siRNAs (Fig. 4B). Interestingly, the decrease in expression of HAI-1 or HAI-2 by their respective siRNAs did not completely inhibit the BMP-mediated effect, which may be due to a partial redundancy in their actions. To study this further, we attempted to simultaneously knockdown HAI-1 and HAI-2 in the NPCs but this proved to be rather difficult and gave variable results (data not shown).

**Figure 5:**
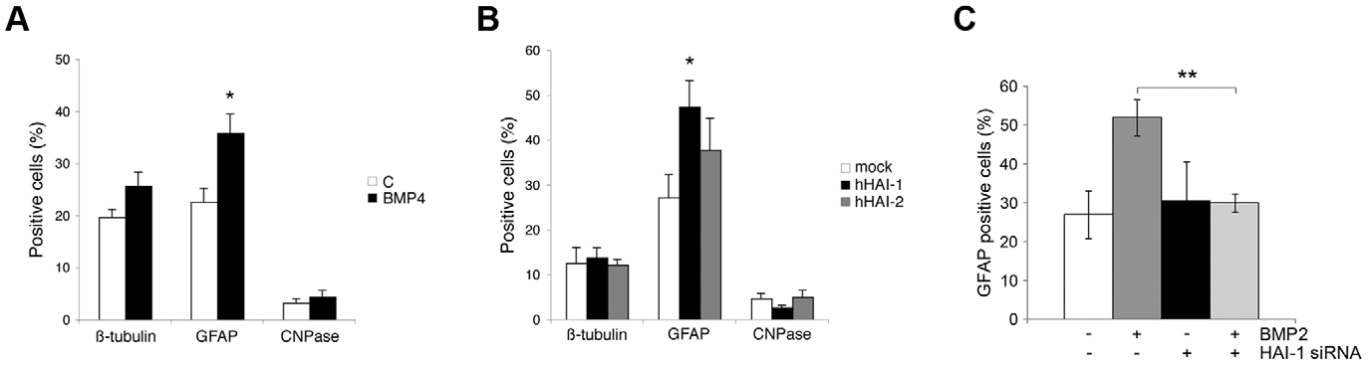
Effects of BMPs and HAI-1 and HAI-2 on cell differentiation. (**A**) NPCs were isolated from developing striatum of E17 old rats and stimulated with 25 ng/ml BMP-4 for 4 days followed by incubation without EGF to induce cell differentiation (see Methods). The relative numbers of neurons (ß-tubulin), astrocytes (GFAP) and oligodendroglial (CNPase) cells were identified using specific antibodies. Note an increase in number of GFAP-positive cells by BMP-4. Values are means ± SEM, n = 4. *p<0.05 for BMP-4 vs. C. (**B**) NPCs were transfected with 5 µg of HAI-1 or HAI-2 expression plasmids and cell differentiation was induced for 5 days. The number of specific cell types was determined by immunostaining. Note an increase in number of GFAP-positive cells by HAI-1. Values are means ± SEM, n = 4. *p<0.05 for HAI-1 vs. control. (**C**) NPCs were transfected with HAI-1 siRNA and stimulated with BMP-2 for 24 h and cell differentiation was induced for 5 days. BMP-2 increased the number of GFAP-positive cells and this effect was inhibited by the downregulation of HAI-1. Values are means ± SEM, n = 3. **p<0.01 for BMP-2+HAI-1 siRNA vs. BMP-2 alone.

We then analyzed whether HAI-1 and HAI-2 exert effects on cell differentiation, a process that is also influenced by BMPs (reviewed in [15]). Removal of the mitogen EGF from the incubation medium induces cell differentiation of NPCs, with the appearance of neuroblasts and glial cells, such as astrocytes and oligodendrocytes [7], [17], [18]. In line with previous results, stimulation with BMP-4 or BMP-2 increased the number of glial fibrillary acidic protein (GFAP)-positive cells in the NPC cultures (Fig. 5A and Fig. 5C). Similarly, overexpression of HAI-1 significantly increased the number of GFAP-positive cells in these cultures (Fig. 5B). There was a trend towards an increase of glial cell number also by using HAI-2, but the effect was not statistically significant (Fig. 5B). We therefore focused on HAI-1 and showed that downregulation of HAI-1 by siRNA led to a significant decrease in the number of GFAP-positive cells induced by BMP-2 (Fig. 5C). Taken together, these findings show that HAI-1 is essentially involved in glial cell differentiation, and that the downregulation of HAI-1 can partly counteract the effect of BMPs on glial cell formation.

### BMPs and BMP Receptors are Expressed in the Neuroepithelium

**Figure 6:**
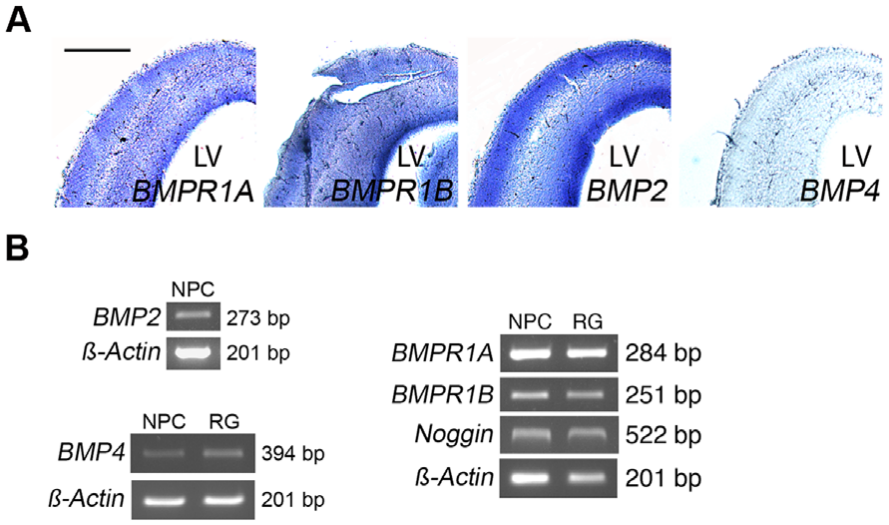
Expression of BMPs and BMP type-I receptors in developing neuroepithelium and in cultured progenitor cells. NPCs were isolated from developing striatum of E17 old rats and Radial glial (RG) cells were derived from mouse embryonic stem (ES) cells as described in Methods. (**A**) *In situ* hybridization was done using E17 old rat dorsal cortex and digoxigenin-labeled probes as described in Methods. Note expression of BMPR-IA and BMPR-IB receptors. BMP-2 was strongly expressed in the neuroepithelium of dorsal cortex. Scale bar, 500 µm. (**B**) RT-PCR was done as described in Methods. BMP-4, BMPR-IA, BMPR-IB as well as Noggin are expressed by cultured rat NPCs isolated from the striatal anlage, and by RG cells derived from mouse ES cells. For BMP-2 only NPCs were studied.

Subsequently we studied the expression of the BMPs and their receptors in the developing neuroepithelium using *in situ* hybridization and sections from E17-old rat dorsal cortex. Data showed that BMP-2 was highly expressed in the neuropeithelium at this developmental stage, whereas BMP-4 expression was rather low (Fig. 6A). Using RT-PCR, BMP-4 transcripts were detected in cultured NPCs isolated from developing rat E17-old striatum and in radial glial cells derived from mouse ES cells (Fig. 6B). The BMPR-IA and BMPR-IB receptors, as well as Noggin that is a BMP inhibitor, are all expressed in the developing neuroepithelium and in the cultured progenitor cells (Fig. 6A and 6B). The presence of both the BMPs and Noggin in the progenitor cells indicate that the BMP signaling is tightly regulated in the NPCs.

### HAI-1 Overexpression Decreases NPC Proliferation and Promotes Glial Cell Formation *In Vivo*

**Figure 7:**
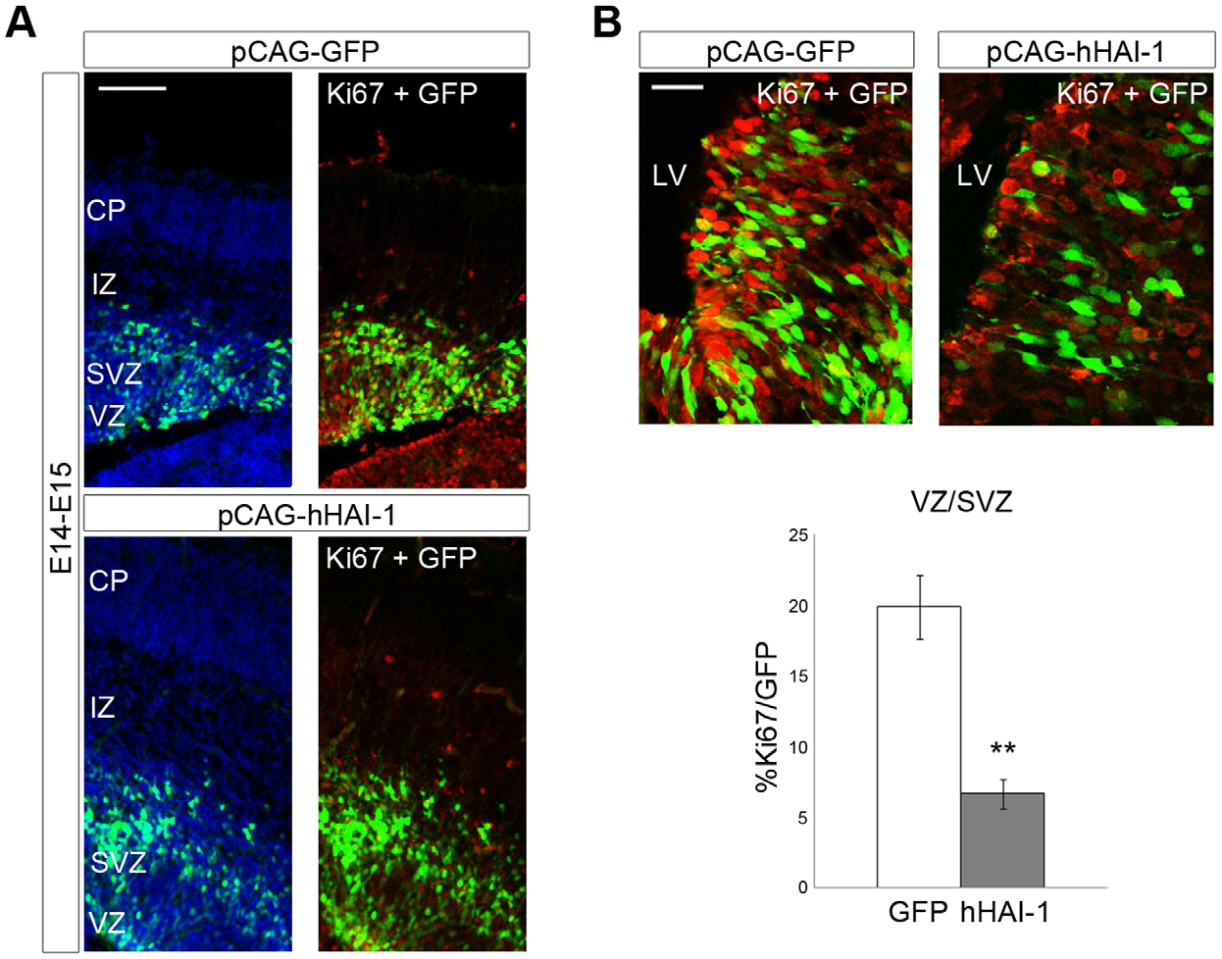
Expression of HAI-1 in developing E14-old rat neuroepithelium reduces cell proliferation. E14 old mice embryos were electroporated as described in Methods using 1 µg pCAG-GFP control plasmid alone or a combination of 0.75 µg pCAG-human HAI-1and 0.25 µg pCAG-GFP. E15-old Brains from E15-old mice were processed as described in Methods. (**A**) E15 brain sections were immunostained with anti-Ki67 antibodies (red). The number of double stained cells in the ventricular and subventricular zones was counted and expressed as a percentage of total GFP-positive cells (green). Upper panel, injection of GFP control plasmid. Lower panel, HAI-1 plasmid. Scale bar, 100 µm. CP, cortical plate; IZ, intermediate zone; SVZ, subventricular zone; VZ, ventricular zone. (**B**) Higher magnification. Scale bar, 50 µm. Left panel, control. Right panel. HAI-1 plasmid. Quantification below. Values are means ± SEM, n = 6. **p<0.01 for HAI-1 vs. GFP. LV, lateral ventricle.

**Figure 8:**
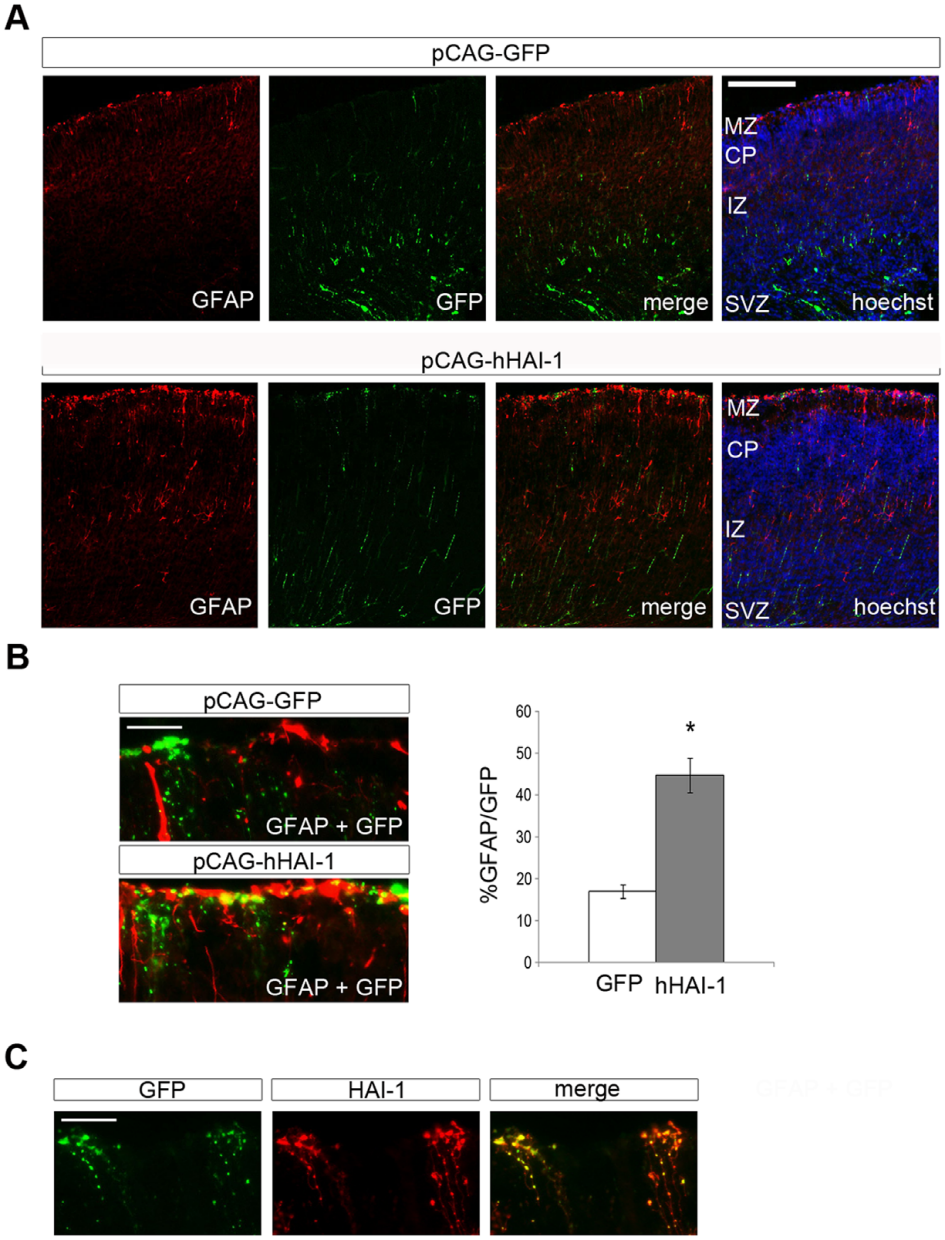
Expression of HAI-1 in developing E17-old rat brain promotes glial cell fate. E17 old mice embryos were electroporated as described above and the brains processed at postnatal day 1 (P1). (**A**) P1-old sections were immunostained with anti-GFAP antibodies (red). The number of double stained cells in the marginal zone was counted and expressed as a percentage of total GFP-positive cells (green). The merged pictures show coexpression of GFAP and GFP. Upper panel, injection of GFP control plasmid. Lower panel, HAI-1 expression plasmids. Scale bar, 100 µm. MZ, marginal zone; CP, cortical plate; IZ, intermediate zone; SVZ, subventricular zone; VZ, ventricular zone. (**B**) Higher magnification. Scale bar, 25 µm. Quantification to the right. Values are means ± SEM. n = 3. *p<0.05 for HAI-1 vs. GFP. (**C**) Higher magnification shows colozalization of HAI-1 and GFP stained cells in the MZ. Scale bar, 25 µm.

To study whether the regulation of NPCs by HAI-1 observed *in vitro* is also observed *in vivo,* we performed *in utero* electroporation in developing mice using an expression vector for HAI-1. Using E14 old mice for injections, we observed that the number of Ki67 positive cells in HAI-1 injected mice day E15 was drastically decreased in the neuroepithelium compared with mice injected with control GFP vector (Fig. 7). At this stage mainly neuroblasts are formed, while gliogenesis is robust during later development. We observed that using E17-old mice that the overexpression of HAI-1 *in utero* led to an increase in the number of GFAP-positive cells in the marginal zone when analyzed at day P1 (Fig. 8). Together these results show that HAI-1 can both reduce cell proliferation and increase gliogenesis in developing NPCs in line with the data obtained in cell culture. The results also show that the effect of HAI-1 overexpression in brain *in vivo* is dependent on the developmental stage of the NPCs in the neuropithelium reflecting probably their inherent capacity to divide or to differentiate into glial cells.

## Discussion

The present study shows that the cell surface-associated serine protease inhibitors, HAI-1 and HAI-2 regulate the behavior of NPCs in the developing brain. Particularly, HAI-1 was found to influence cell division and differentiation of NPCs into glial cells. This notion is supported by *in vitro* and *in vivo* data and suggests that HAI-1 is an important molecule in the regulation of NPCs. Cell surface-expressed protease inhibitors like HAI-1 may constitute a novel signaling system in the NPCs different from cell adhesion molecules and integrins to influence NPCs. As shown here for BMP-2 and BMP-4, HAI-1 may act in conjunction with growth factors to orchestrate the responses of NPCs towards cell proliferation or differentiation.

HAI-1 encoded by the *Spint1* gene was originally identified as an inhibitor for hepatocyte growth factor activator (HGFA) [4], [19]. Studies of mouse development have shown that HAI-1 gene-deleted animals die *in utero* due to placental dysfunctions and basement membrane failures [20], [21]. Gene deletion of HAI-2 encoded by the *Spint2* gene [22] also resulted sin evere developmental defects and embryonic lethality [23], [24].

HAI-1 and HAI-2 are structurally related protease inhibitors having two extracellular Kunitz inhibitor domains, KD1 and KD2 that are important for activity [25]–[27]. Few data are so far available on the function of HAI-1 or HAI-2 in brain tissue. HAI-1 is expressed by human astrocytes and in cell lines derived from human brain gliomas [28], [29]. HAI-1 also increases the tumorigenicity of glioblastoma cells *in vivo* but reduces the invasive capabilities of the same cells [30]. HAI-2 mRNA was reported to be expressed in adult human brain but not in fetal brain using Northern blotting [22]. HAI-2 expression was reduced in glioblastoma cells *in vitro* [31], and HAI-2 may act as a tumor suppressor as shown previously in pediatric medulloblastomas [32].

We observed that HAI-2 displayed similar activities to HAI-1 in reducing NPC proliferation. In contrast, HAI-2 was less active in regulating glial cell differentiation, suggesting that these serine protease inhibitors may have partly different substrates in the NPCs. HAI-2 was recently shown to be expressed in the early neural tube in conjunction with the serine protease, matriptase [24]. As *bona fide* protease inhibitors, HAI-1 and HAI-2 have the capacity to inhibit different proteases at the cell surface. Recently it was shown that neural progenitor cells derived from mouse embryonic stem cells express matriptase [33]. We have preliminary findings showing that rat NPCs express matriptase both in the developing neuroepithelium and *in vitro* (unpublished). Collectively this data indicates that the cell surface serine protease inhibitors HAI-1 and HAI-2 may affect brain development and NPCs via inhibiting serine proteases, such as matriptase. We are currently investigating this in more detail.

Given that HAI-1 was described as an inhibitor of HGFA [4], [19], we also studied whether HGF might be involved in the action of the HAI-1 in the NPCs. However, we observed that the HGF receptor, c-Met was not expressed by the NPCs to any significant extent nor did the addition of HGF influence NPCs under the present conditions. Previously, HGF has been shown to influence different neuronal and progenitor cell populations in the brain [34], [35]. It is likely that HGF may influence NPCs at a later stage of development affecting for example cell migration as shown for embryonic stem cell-derived progenitor cells in culture [33].

As shown in Fig. 3 the study of the effect of different growth factors revealed that BMP-2 and BMP-4 increase the expression of HAI-1 and HAI-2 in the NPCs. BMP signaling in target cells take place through activation of a heteromeric complex of type-I and type-II serine/threonine kinase receptors [10]. Previous studies have shown that different BMP receptors are expressed in the developing and adult brain [36]–[38]. BMP-2 and BMP-4 bind the BMPR-IA and BMPR-IB receptors and activate Smad1/5/8 that forms a heteromeric complex with Smad4 [10] that translocates to the nucleus to regulate the expression of various genes, including the transcription factor GATA2 [39]. Putative binding sites for GATA2 are present in the promoter regions of HAI-1 and HAI-2 [40] suggesting that GATA2 may be involved in the BMP-mediated increases in HAI-1 and HAI-2 expression observed here.

Of the BMPs studied here, BMP-2 was more prominently expressed in the developing neuroepithelium as shown in sections of dorsal cortex, whereas the levels of BMP-4 were low as determined by *in situ* hybridization. In the cultures, developing rat NPCs as well as mouse RGs express BMP-4 as shown using PCR. These cells also expressed the BMPR-IA and BMPR-IB receptors, which mediate the effects of both BMP-2 and BMP-4 on target cells. In line with this we observed that BMP-2 and BMP-4 had similar effects on NPC proliferation and glia cell induction and both also increased HAI-1 and HAI-2 expression. It remains to be studied whether BMP-4 is expressed in significant amounts in the developing striatum and which progenitor populations might be targets for this growth factor and for BMP-2 in developing brain *in vivo.*

Previously BMPs were shown to regulate cell death, cell proliferation, neurogenesis and gliogenesis in brain tissue [15]. The activities of BMPs are controlled by inhibitors like Noggin, and are influenced by other growth factors that may act in concert with the BMPs in the neurogenic niche [41], [42]. Recently it was shown that BMP signaling regulates the quiescence state of neuronal progenitor cells in the subgranular zone of adult hippocampus [43]. In particular, the activation of BMPR-IA receptors by BMP-2 or BMP-4 was shown to reduce cell proliferation of the hippocampal NPCs [43], but the downstream effectors were not further delineated. We show here that these BMPs regulate cell proliferation of embryonic NPCs partly via the upregulation of HAI-1. HAI-1 in turn influences cell signaling events and the behavior of NPCs. It remains to be determined whether the BMPs also increase HAI-1 in the subgranular zone and whether this can contribute to the regulation of the neurogenic niche in adult brain. In addition the identity of putative downstream proteases regulated by HAI-1 and HAI-2 in the NPCs and their role in cell proliferation and differentiation of these cells warrant to be studied more in the future.

Taken together these results show that BMP-2 and BMP-4 increase HAI-1 and influence cell proliferation and glial cell differentiation of developing NPCs. This finding may be of general relevance as BMP-2 and BMP-4 may regulate the cell-surface protease inhibitors also in other stem cell types and in tumor cells with effects on cell growth and differentiation. The data also suggests that modulation of cell-surface expressed HAI-1 and its downstream proteases in the NPCs may be of value for different cell therapies in the brain.

## Materials and Methods

### Animals

Wistar rats were obtained from Harlan (Horst, The Netherlands) and NMRI mice from our own breeding. All animal experiments were performed in accordance with the European Communities Council Directive (86/609/EEC) and approved by the Experimental Animal Ethics Committee of the National Laboratory Animal Center, Finland.

### Cell Culture

NPCs were prepared from striatum of embryonic day 17 to 18 (E17–18) old rats and the cells were plated in Corning Suspension Culture dishes (5×10^6^ cells per 10 cm dish; Corning Incorporated, Corning, NY, USA) and cultivated in a serum-free DMEM/F12 medium (Gibco, Invitrogen Carlsbad, CA, USA) in the presence of B27 supplement (Gibco) and 20 ng/ml epidermal growth factor (EGF) (Peprotech, Rocky Hill, NJ, USA) at +37°C in 5% CO_2_ [7], [17], [18], [44]. Cells were grown for 5 days in culture before passaging and then used for the experiments. Recombinant BMP-2, BMP-4, Wnt3a and Noggin (all from R&D Systems, MN, USA), HGF (Peprotech, Rocky Hill, NJ, USA) and the c-Met inhibitor, SU11274 (Calbiochem, San Diego, CA, USA) were added at the indicated concentrations. TGFβ was a kind gift from Dr J Keskioja. Hippocampal neurons were prepared and cultured as described previously [45], [46].

### Cell Proliferation

NPCs were dissociated and plated onto 50 µg/ml poly-DL-ornithine-coated (Sigma) 24–well culture dishes (150000 cells per well; Cellstar, Greiner Bio-one) for 2 h and fixed with 4% paraformaldehyde (PFA) for 20 min, washed and subjected to immunocytochemistry using anti-mouse Ki67 antibody (1∶500; BD Biosciences, Franklin Lakes, NJ, USA). Alternatively, 10 µM BrdU (Sigma, St. Louis, MO, USA) was added to the cells for 24 h, and DNA was denaturated, and immunostaining performed using anti-rat BrdU (1∶200; Sigma) antibody as described previously [7], [47], [48]. The number of Ki67-, or BrdU-positive cells was counted using microscopy in four non-overlapping fields per coverslip. Experiments were repeated at least three times.

### Cell Differentiation Assay

Dissociated cells were plated onto 50 µg/ml poly-DL-ornithine-coated 24-well culture dishes (100 000 cells per well; Cellstar), and incubated for 5 days at +37°C. Cells were fixed as above, washed with PBS, and subjected to immunocytochemistry using ßIII-tubulin (1∶1000; Covance Research Products Inc., Denver, PA, USA), GFAP (1∶500; Sigma) and CNPase (1∶1000; Sigma) antibodies. The number of immunopositive cells were counted using microscopy in four non-overlapping fields per coverslip. Experiments were repeated at least three times.

### Radial Glia Cells

Mouse embryonic stem (ES) cells (strain R1) were cultured on mitomycin-C-inactivated mouse embryonic fibroblast (mEF) cells or on 0,2% gelatin. ES cells were cultivated in knockout-DMEM medium (Invitrogen) containing 15% fetal calf serum (FCS), leukemia inhibitory factor (LIF; 1000 U/ml, Merck, Germany), non-essential amino acids and ß-mercaptoethanol. Medium was changed every day and cells passaged at 70–80% confluency. ES cells without feeders were split every second day and then used for embryoid body (EB) formation. 3×10^6^ ES cells were plated on fibroblast-free, ultra-low-attachment dishes in culture medium with 10% FCS and without LIF. EBs were cultured for 8 days with medium change every other day. 5 µM retinoic acid (Sigma) was added to the EB cultures for the last 4 days and cells were dissociated using trypsin and plated onto poly-DL-lysine/laminin-coated dishes (1.5×10^5^ cells per cm^2^) in N2B27 medium as described [49]. The RGs formed were incubated for 2 h and analyzed further as indicated.

### Expression Studies

Expression vectors (pCIneo) for human HAI-1 (pCI-hHAI-1), truncated human HAI-1 (pCI-hHAI-1dTM), human HAI-2 (pCI-hHAI-2) and truncated human HAI-2 (pCI-hHAI-2dTM) have been described before [26], [27], [32]. Plasmids were transfected into NPCs using Amaxa Nucleofector (Lonza Cologne GmbH, Germany), and 24 h after transfection the medium was replaced, and cells were further cultured in the presence of 0.6 mg/ml of G418 (Sigma). G418-resistant cells were selected, screened for HAI-1 and HAI-2 expression and used in studies of cell proliferation, differentiation and for immunoblotting. Mock-transfected EGFP expressing cells were used as controls. For *in utero* electroporation, human HAI-1 cDNA was digested from pCI-hHAI-1 vector with the desired restriction enzymes and subcloned into the *Eco*RI/*Sma*I site of a pCAG expression vector (Addgene, Cambridge, MA, USA).

### siRNA Experiments

100 nM HAI-1 or HAI-2 siRNA (Applied Biosystems/Ambion, Austin, TX, USA) was transfected into NPCs using the Rat NSC Nucleofector Kit as described [47], [48]. Scrambled siRNA with no target was used as control. Cells were incubated for 3 days and studied further as indicated. Efficacy of downregulation was analyzed by immunoblotting or using RT-PCR.

### Reverse Transcriptase (RT)-PCR

Total RNA was extracted from cultured cells using the GenElute Mammalian total RNA kit (Sigma). cDNA was made using the reverse transcriptase kit (Stratagene) [50]. Primers for the different genes and conditions used for PCR are given in Table 1. The PCR was run for 35 cycles and with a final run at 72°C for 10 min.

**Table 1:** Specific primers, product size and annealing temp for PCR.

Gene	Forward primer, 5′–3′	Reverse primer, 5′–3′	Size(bp)	Temp
HAI-1	GAGCAGAACTTCGTGTG	GTAGTTGTTCTTGTTGCC	500	59°C
HAI-2	ATGAAGAATACTGTGTC	GCTGCTCCTTGTCATC	347	45°C
c-Met	ATAGAGTGGAAGCAAGC	GCACTTACAAGCCTATC	229	58°C
BMP-2	TCCATCACGAAGAAGCC	ACTGACTTGTGTTCTGAG	272	50°C
BMP-4	AGCCAACACTGTGAGGAG	TGTCTGGTGGAGGTGAGT	393	54°C
BMPRIA	AGCCTGTCTGTTCATCATT	CAGAGCCTTCATACTTCAT	283	54°C
BMPRIB	TACCTCATCACAGACTATC	CTAATGAACTTGACAGCCA	250	48°C
Noggin	AGCACTATCTACACATCCG	CACAGACTTGGATGGCTTA	511	62°C
β-actin	CACACTGTGCCCATCTATGA	CCATCTCTTGCTCGAAGTCT	201	62°C

### Quantitative (q)PCR

Total RNA was prepared as above. cDNA for HAI-2 qPCR was made using the Superscript® VILO™ cDNA Synthesis kit (Invitrogen). For HAI-1 qPCR, gene-specific cDNA was prepared with HAI-1 and GAPDH primers, 5′-AAGCTTCGGTGTCCAACA-3′ and 5′-AAGGTGGAAGAATGGGAG-3′, respectively, using the RevertAid Premium reverse transcriptase (Thermo Scientific, Waltham, MA, USA). qPCR analysis was performed using the comparative C_T_ method and the Power SYRB® Green PCR Master Mix (Applied Biosystems) essentially as described [50], [51] or the LightCycler® 480 SYBR Green I Master Mix (Roche, Basel, Switzerland). Assays were performed in triplicate including water as a negative control, and using the ABI PRISM 7000 sequence detection system (Applied Biosystems) and the following conditions: 95°C for 10 min and 46 cycles of 95°C for 20 s and 60°C for 1 min or using the LightCycler® 480 II system (Roche) with following conditions: 95°C for 10 min and 40–42 cycles of 95°C for 10 s, 60°C for 15 s, 72°C for 5 s followed by 72°C for 1 min. Primers for HAI-1 were: fwd, 5′-GCCAGCATCTCTACGGTCT-3′ and rev, 5′-ACGGCAGCTCGGTCTCA-3′. Primers for HAI-2 were: fwd, 5′-GGCTGTGAGGGAAATGGTAA-3′ and rev, 5′-ACCATCAATGGTGTTCTCAGTG-3′.

### Immunoblotting

NPCs and hippocampal neurons were homogenized in ice-cold RIPA buffer (150 mM NaCl, 1% Triton-X-100, 0,25% sodium deoxycholate, 1% SDS, and 50 mM Tris-HCl, pH 7.4, 1 mM EDTA) supplemented with protease inhibitor cocktail (Roche). In some experiments, phosStop solution (Roche) was added to inhibit phosphatases. Protein concentration was determined using the DC Protein Assay (BioRad, Finland), and equal amounts of protein were separated by SDS-PAGE and transferred onto a nitrocellulose filter (Hybond-C Extra; Amersham, England). Filters were incubated for 1 h in 50 mM Tris-HCl, pH 7.5, 150 mM NaCl, 0.1% Tween 20, and using 5% skimmed milk or bovine serum albumin, followed by incubation overnight at +4°C with primary antibodies. These included anti-rabbit HAI-1 (diluted 1∶2000, Santa Cruz), anti-rabbit polyclonal mHAI-1 (1∶200, ref [20]), anti-rabbit polyclonal mHAI-2 (1∶400, ref [27]), or anti-mouse monoclonal hHAI-2 2N9 (1∶300, ref [28]), anti-rabbit Erk1/2 and P-Erk1/2 (1∶1000, Cell Signaling Technology, Inc., Danvers, MA, USA), anti-mouse CyclinD1 (1∶750), anti-goat HGFA (1∶500), and anti-rabbit HGF (1∶300, Santa Cruz, CA, USA) or anti-mouse c-Met (1∶1000, Cell Signaling). Filters were washed and exposed to horseradish peroxidase-conjugated secondary antibodies (diluted 1∶2500, Pierce), followed by detection using the enhanced chemiluminescent method (Pierce). Filters were stripped for 20 min at room temperature (RT) (0.1M glycine) and reprobed using anti-β-actin (1∶5000, Sigma) antibody.

### Immunocytochemistry

Cells plated on poly-DL-ornithine (Sigma) coated coverslips were fixed with 4% PFA for 20 min, washed with PBS and incubated for 1 h with 3% BSA in 0.1% Triton-X-100 and PBS (PBS-T), followed by incubation at +4°C overnight with primary antibodies, including anti-goat mHAI-1 (1∶100, R&D Systems), anti-goat mHAI-2 (1∶20, R&D Systems), anti-mouse Ki67 (1∶500), anti-rabbit p-Smad1/5/8 (1∶400, Cell Signaling) or anti-mouse nestin (1∶1000; Millipore). Cells were washed with PBS-T, and appropriate secondary Alexa 488-conjugated anti-mouse or anti-rabbit, and Alexa 594-conjugated anti-rabbit or anti-goat antibodies were added for 1 h. Cells were counterstained for 5 min using Hoechst 33342 (4 µg/ml, Sigma), washed with 0,1 M Tris-HCl (pH 7,4), and mounted using Fluoromount gel mounting medium (Sigma).

### Immunohistochemistry of Paraffin and Frozen Sections

E17 and P1 old rat brains were embedded in paraffin and cut at the coronal plane using a microtome [46], [52]. 5–7 µm thick sections were dewaxed in xylane and rehydrated in decreasing ethanol series and water. Antigen retrieval was performed by boiling sections 3 times at 95°C for 5 min in 10 mM citrate, pH 6.0. Sections were cooled for 10 min, washed with PBS, and blocked with 5% BSA in PBS-T. Primary anti-goat mHAI-1 (1∶20), anti-goat mHAI-2 (1∶20), anti-mouse Ki67 (1∶500) and anti-mouse nestin (1∶1000) in 3% BSA in PBS-T were added overnight at +4°C. After washing, secondary anti-goat Alexa 594-conjugated and anti-mouse Alexa 488-conjugated antibodies (Invitrogen) were added for 1 h. The sections were briefly washed, counterstained with Hoechst 33342 and mounted as above.

E15 or P1 mouse brains were mounted into Tissue-Tek (Sakura Finetek, Netherlands) and frozen on dry ice. Brains were cut into 16 µm coronal sections with a cryotome. After melting, cryosections were fixed in ice-cold acetone-methanol (1∶1) and rinsed with PBS. Sections were subsequently subjected to antigen retrieval for 3×3 min, and washed with PBS-T before blocking with 5% BSA/PBS-T. Anti-mouse Ki67 (1∶500), anti-rabbit GFAP (1∶500) or anti-rabbit HAI-1 antibody (1∶50, Santa Cruz) were added, and the sections were incubated overnight at +4°C. After washing, secondary Alexa 594-conjugated anti-mouse or Alexa 594-conjugated anti-rabbit antibodies were added for 1 h. The sections were washed, counterstained with Hoechst 33342 and rinsed with 70% ethanol before mounting with 1,4-Diazabicyclo[2.2.2]octane (DABCO; Merck) diluted in Mowiol mounting media (Sigma).

### *In Situ* Hybridization

Whole mount *in situ* hybridization was performed essentially as described using E17 rat brain tissues that were fixed overnight at 4°C using 4% PFA. Brains were embedded in 5% agarose and 40–80 µm thick sections were cut using the Leica VT 1000S Vibratome (Leica). Sections were incubated at 37°C for 30 min with 0.5 mg/ml Proteinase K following hybridization using digoxigenin (DIG)-labelled cRNA probes and the InSituPro automate (Intavis). Antisense and sense riboprobes were synthesized from the following linearized plasmids: pGEZ-3Z (BMPR-IA), pGEM-4Z (BMPR-IB) or pGEM (BMP-2 and BMP-4) and using T7 or SP6 polymerases. Sections were hybridized for 16 h at 65°C and mounted.

### *In Utero* Electroporation

E14 or E17 mouse embryonic brains were injected with 1 µg pCAG-GFP control plasmid alone (1 µg/µl) or with a combination of pCAG-HAI-1 and pCAG-GFP (0.75 µg/µl and 0.25 µg/µl, respectively). In brief, mice were anesthetized with isoflurane (induction 4,8%, maintenance 2–3%), and the depth of sleep was monitored by the rate of breathing. A midline incision was made in the abdominal skin and muscle. Uterine horns were exposed and hydrated during the surgery using 37°C warm phosphate buffered saline (PBS). Plasmids supplemented with 0.01% Fast Green (Sigma) were injected with a glass capillary needle through the uterine wall into the lateral ventricle and the brains were electroporated with forceps-type electrodes (CUY650P3, Sonidel) using a square-wave pulse generator (CUY21SC, Sonidel) in an dorsal to lateral direction [53]. Embryos received 5 pulses with voltage of 50 V, lasting for 0.05 s and with a pulse interval of 0.95 s. The uteri were carefully placed back and the abdominal cavity was filled with warm PBS and sutured. Mice received Temgesic (4.5 µg/50 g) to reduce pain and were kept on a heated pad during the whole operation and during recovery. Brains were collected at E15 or P1 and fixed with 4% PFA overnight at +4°C. After washes with PBS, the brains were immersed with 30% sucrose in PBS and analyzed further as indicated.

### Image Analysis and Statistics

Fluorescent images were taken with the Leica DM 4500B microscope using N Plan 10×/0.25 objective, the Zeiss Axio Observer Z1 inverted microscope using N Achroplan 10×/0.25 and EC Plan-Neofluar 40×/0.75 objectives or Plan-Apochromat 63×/1.4 oil objective or with the Zeiss LSM 510 Meta confocal microscope using Plan-Neofluar40×/1.3 or Plan-Apochromat 63×/1.4 oil objectives. Brightfield images were taken with the Leica DM 4500B microscope using N Plan 2.5×/0.07 objective. Leica Application Suite Version 2.3.1 R1, AxioVision Release 4.8.1 and LSM software release 3.2 softwares were used for image acquisition. Adobe Photoshop was used for adjustment of brightness and/or contrast of images. Statistical analyses were performed using Student’s *t*-test. Values are given as means ± SEM and p<0.05 was considered as statistically significant.

## Supporting Information

Figure S1 **Hepatocyte growth factor signaling in the NPCs.** NPCs from E17 old rat brains were treated and analyzed as indicated below. **(A)** Immunoblots. Left, Hepatocyte growth factor activator (HGFA) protein is expressed in NPCs but the levels are not influenced by HAI-1 or HAI-2 overexpression. Right, HGF is expressed mainly in proliferating (P) and less in differentiating (D) NPCs. The 90 kDa band is the nascent single chain, and the 69 kDa the active form of HGF. ß-actin was used as a control. Typical experiment is shown and was repeated three times. **(B)** Analyzes of the c-Met receptor for HGF by RT-PCR (left panel) and immunoblots (right panel). Lysates from hippocampal neurons (HC) were used as controls. c-Met was expressed in HC but was not detectable in NPCs using these Methods. **(C–D)** NPCs were incubated in the presence of 1 µM c-Met inhibitor, SU11274 (C) or after addition of 20 ng/ml HGF (D). There was no change in the number of BrdU-positive cells by these treatments. **(E)** 20 ng/ml HGF was added to NPCs in which HAI-1 or HAI-2 were downregulated using siRNAs as described in Methods. The number of dividing NPCs was determined using BrdU labeling. Note an increase in cell proliferation after downregulation of HAI-1 and HAI-2 but no effect of HGF. Values are means ± SEM, n = 3. *p<0.05 for HAI-siRNAs vs. control. N.s, not significant.
(TIF)
