# Supplementary figures and images for: Hepatocyte Growth Factor Activator Inhibitor-1 Is Induced by Bone Morphogenetic Proteins and Regulates Proliferation and Cell Fate of Neural Progenitor Cells
Source: PLoS One. 2013 Feb 7;8(2):e56117. doi: 10.1371/journal.pone.0056117 (PMC3567048; doi:10.1371/journal.pone.0056117)

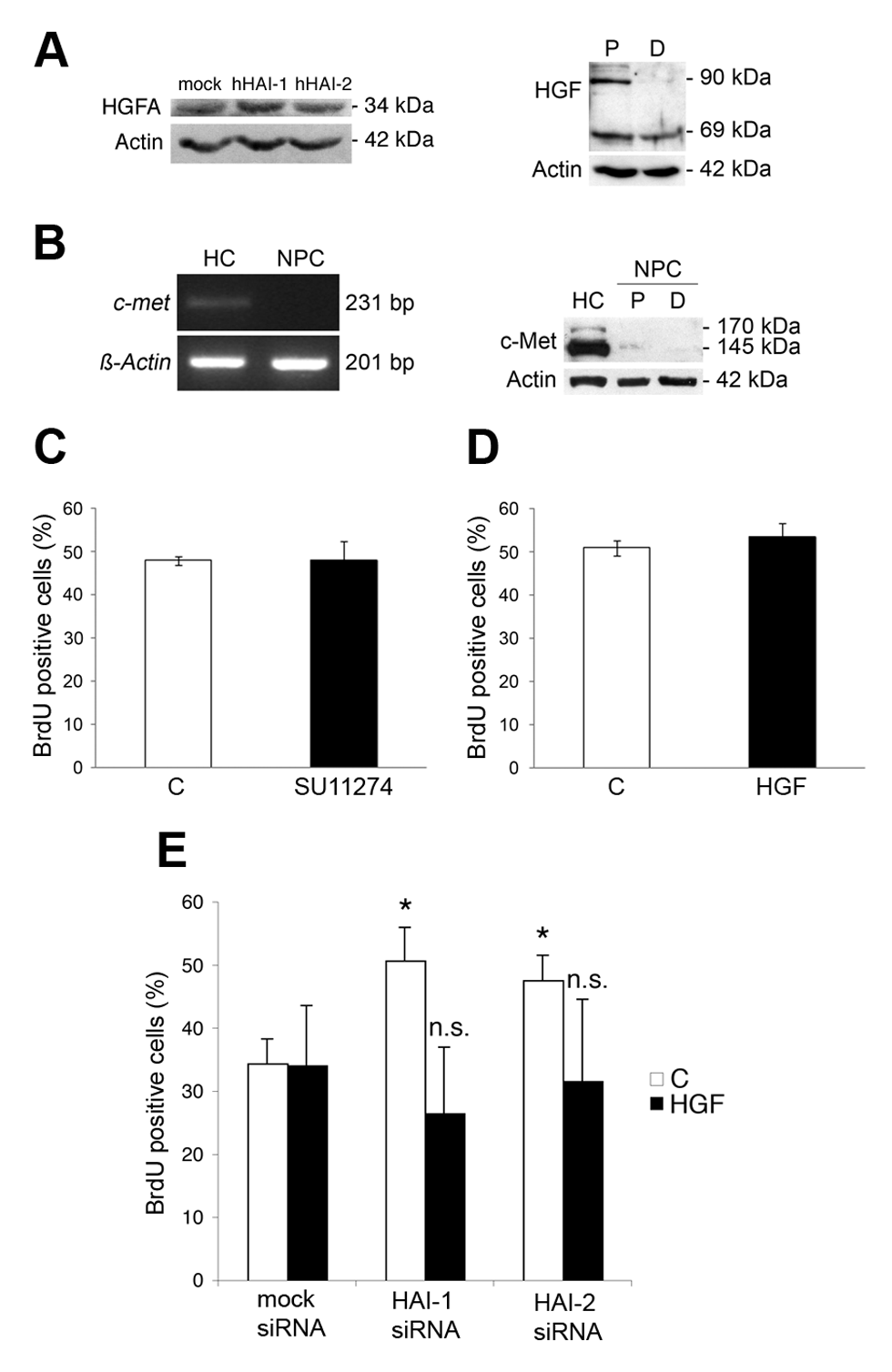

Supplement: Figure S1 — Hepatocyte growth factor signaling in the NPCs. NPCs from E17 old rat brains were treated and analyzed as indicated below. (A) Immunoblots. Left, Hepatocyte growth factor activator (HGFA) protein is expressed in NPCs but the levels are not influenced by HAI-1 or HAI-2 overexpression. Right, HGF is expressed mainly in proliferating (P) and less in differentiating (D) NPCs. The 90 kDa band is the nascent single chain, and the 69 kDa the active form of HGF. ß-actin was used as a control. Typical experiment is shown and was repeated three times. (B) Analyzes of the c-Met receptor for HGF by RT-PCR (left panel) and immunoblots (right panel). Lysates from hippocampal neurons (HC) were used as controls. c-Met was expressed in HC but was not detectable in NPCs using these Methods. (C–D) NPCs were incubated in the presence of 1 µM c-Met inhibitor, SU11274 (C) or after addition of 20 ng/ml HGF (D). There was no change in the number of BrdU-positive cells by these treatments. (E) 20 ng/ml HGF was added to NPCs in which HAI-1 or HAI-2 were downregulated using siRNAs as described in Methods. The number of dividing NPCs was determined using BrdU labeling. Note an increase in cell proliferation after downregulation of HAI-1 and HAI-2 but no effect of HGF. Values are means ± SEM, n = 3. *p<0.05 for HAI-siRNAs vs. control. N.s, not significant. (TIF) [file pone.0056117.s001.tif]
